# Unveiling biosynthetic potential of an Arctic marine-derived strain *Aspergillus sydowii* MNP-2

**DOI:** 10.1186/s12864-024-10501-0

**Published:** 2024-06-17

**Authors:** Zhiyang Fu, Xiangzhou Gong, Zhe Hu, Bin Wei, Huawei Zhang

**Affiliations:** School of Pharmaceutical Sciences, Zhejiang University of Technology, 310014, Hangzhou, China

**Keywords:** Polar microorganisms, *Aspergillus Sydowii*, Whole-genome sequence, Biosynthetic gene cluster, antiSMASH, Molecular networking

## Abstract

**Background:**

A growing number of studies have demonstrated that the polar regions have the potential to be a significant repository of microbial resources and a potential source of active ingredients. Genome mining strategy plays a key role in the discovery of bioactive secondary metabolites (SMs) from microorganisms. This work highlighted deciphering the biosynthetic potential of an Arctic marine-derived strain *Aspergillus sydowii* MNP-2 by a combination of whole genome analysis and antiSMASH as well as feature-based molecular networking (MN) in the Global Natural Products Social Molecular Networking (GNPS).

**Results:**

In this study, a high-quality whole genome sequence of an Arctic marine strain MNP-2, with a size of 34.9 Mb was successfully obtained. Its total number of genes predicted by BRAKER software was 13,218, and that of non-coding RNAs (rRNA, sRNA, snRNA, and tRNA) predicted by using INFERNAL software was 204. AntiSMASH results indicated that strain MNP-2 harbors 56 biosynthetic gene clusters (BGCs), including 18 NRPS/NRPS-like gene clusters, 10 PKS/PKS-like gene clusters, 8 terpene synthse gene clusters, 5 indole synthase gene clusters, 10 hybrid gene clusters, and 5 fungal-RiPP gene clusters. Metabolic analyses of strain MNP-2 grown on various media using GNPS networking revealed its great potential for the biosynthesis of bioactive SMs containing a variety of heterocyclic and bridge-ring structures. For example, compound G-8 exhibited a potent anti-HIV effect with an IC_50_ value of 7.2 nM and an EC_50_ value of 0.9 nM. Compound G-6 had excellent in vitro cytotoxicities against the K562, MCF-7, Hela, DU145, U1975, SGC-7901, A549, MOLT-4, and HL60 cell lines, with IC_50_ values ranging from 0.10 to 3.3 µM, and showed significant anti-viral (H1N1 and H3N2) activities with IC_50_ values of 15.9 and 30.0 µM, respectively.

**Conclusions:**

These findings definitely improve our knowledge about the molecular biology of genus *A. sydowii* and would effectively unveil the biosynthetic potential of strain MNP-2 using genomics and metabolomics techniques.

**Supplementary Information:**

The online version contains supplementary material available at 10.1186/s12864-024-10501-0.

## Background

A rich diversity of microorganisms exists in various polar habitats, and these microorganisms have evolved physiological, genetic, and metabolic characteristics adapted to extreme environments under the selection of long-term extreme environmental stresses, and thus have important basic research value and great application potential [1–3]. A growing number of studies have demonstrated that the polar regions have the potential to be a significant repository of microbial resources and a potential source of active ingredients. There were 263 new natural products discovered between 2001 and 2020 that were derived from polar organisms, 134 of which were polar microorganisms [4, 5]. These products covered a wide range of structural types, including alkaloids, macrolides, terpenoids, peptides, and polyketides, and they showed promising biological activities like antibacterial [6], antitumor [7], and antiviral [8] effects. The quantity of intriguing secondary metabolites with polar microbial origins has not altered considerably over the past few years, most likely because it is challenging to adapt polar bacteria to some common culture techniques.

Polar marine microbe-derived natural compounds offer a tremendous amount of potential for utilization as sources of therapeutic agent [9]. As fewer drugs become available, researchers are increasingly focusing on special microbial resources, such as habitat-specific microbes, and have begun to shift away from bioactivity-guided fractionation as the gold standard approach for natural product discovery, instead turning to genomics, metabolomics, and other big data approaches to guide isolation efforts towards uncharted chemical space [10, 11]. Even well-studied organisms have untapped biosynthetic potential because they encode a large number of biosynthetic genes that have not yet been connected to metabolite products [12]. Paulus [13] et al. localized the α-pyrone lagunapyrone biosynthetic gene cluster of a marine origin *Streptomyces* strain by antiSMASH and identified two analogues. Hou [14] et al. successfully identified seven cyclohexadepsipeptides, chrysogeamides A–G, from the coral-derived fungus *Penicillium chrysogenum* using MN. Liu [15] et al. used MN localization to determine two wealthy polycyclic macrolactam ansamycins from *Streptomyces*. Sun [16] et al. successfully obtained 11 omicsnin analogues while identifying a biosynthetic gene cluster for antiviral components by integrating antiSMASH, MN, and other techniques. Here, we generated the arctic marine-derived strain *Aspergillus sydowii* MNP-2 genome using a combination of Nanopore and Illumina sequencing data sets and used Nr, COG, KEGG, CO, Pfam, and some other databases for gene function annotation based on sequence similarity or Motif similarity search. Additionally, to investigate the natural product synthesis ability of strain MNP-2 further, we used the antiSMASH platform to analyze its BGCs and the GNPS platform to establish a molecular network based on LC-MS/MS data to analyze its metabolite profile and associate metabolites with BGCs.

## Methods

### Genome extraction and next-generation sequencing

The fungal strain MNP-2 of *A. sydowii* was derived from Arctic marine sediments (73.8° N 168.9° W) and is preserved at the China Center for Type Culture Collection (CCTCC NO: M 2,022,061). The strain MNP-2 grown on potato dextrose agar (PDA) media was inoculated into 250 mL Erlenmeyer flasks containing 100 mL potato dextrose broth (PDB) medium and shaken for 2 days at 200 rpm and 30 ℃. After centrifugation, the supernatant was removed and washed once with phosphate buffered solution (PBS) to obtain mycelium for storage at -80 ℃. The purity and integrity of the genomic DNA were evaluated using 1% agarose gel electrophoresis and densitometry on comparably sized standards.

The yield and purity of the collected DNA were determined using a NanoDrop 2000 spectrophotometer (Thermo Fisher Scientific, USA) and a Qubit 2.0 fluorometer (Thermo Fisher Scientific, USA). After the DNA samples were tested and qualified, the libraries were built, and after they were finished, the libraries were diluted using Qubit 2.0 for initial quantification, and then the insert fragments of the libraries were tested using Agilent 2100. After the insert fragment size exceeded the expectation, the effective concentration of the libraries was correctly measured using the Q-PCR method to guarantee the libraries’ quality. After the libraries passed the test, they were divided into flow cells based on their effective concentrations and downstream data volume requirements. cBOT was formed into clusters and sequenced using Illumina NovaSeq, Illumina’s high-throughput sequencing platform.

### SMRT sequencing

The qualified samples described in 3.1 were randomly interrupted by Megaruptor for genomic DNA, and large fragments of DNA were enriched and purified using magnetic beads. large fragments were cut and recovered using a BluePippin automated nucleic acid recovery instrument, and after purification, end repair and addition of acid was performed at both ends of the DNA fragments, and the SQK-LSK109 kit. Finally, the DNA library was accurately quantified using Qubit. After library construction, a certain concentration and volume of DNA library is added to the flow cell, and the flow cell is transferred to the Oxford Nanopore PromethION sequencer for real-time single-molecule sequencing.

### Genome assembly

Because raw data may comprise low-quality sequences, joint sequences, and so on, the raw data must be filtered to obtain legitimate data (clean reads or pass reads) and then stored in FASTQ format to ensure the dependability of the information analysis results. SOAPnuke (v2.1.2, https://github.com/BGI-flexlab/SOAPnuke) is used to filter the raw data from next-generation sequencing. The raw data for third-generation sequencing is fast5 files, which are converted to fastq format after base calling with GUPPY, and then filtered to obtain valid data. K-mer values were automatically selected based on the read length and data type. NECAT (v0.0.1, https://github.com/xiaochuanle/NECAT) software was used to correct and splice the genome to obtain the initial splicing results, then Racon (v1.4.11, https://github.com/isovic/ racon) software was used to perform two rounds of error correction on the splicing results based on third generation sequencing data, and then Pilon (v1.23, https://github.com/broadinstitute/pilon) software was used to perform two rounds of next generation sequencing error correction on the initial assembly results after third generation sequencing error correction. The final assembly results were obtained by deduplicating the corrected genomes using purge_haplotigs (v1.1.2, https://github.com/skingan/purg e_haplotigs_multiBAM).

### Gene annotation

Gene structure prediction allows researchers to obtain extensive information about the genome’s gene distribution and structure, as well as vital raw materials for functional annotation and evolutionary study. Gene annotation of the MNP-2 genome was conducted using BRAKER (v2.1.4, https://github.com/Gaius-Augustus/BRAKER) software, which is a combination of GeneMark-ET [17], and AUGUSTUS [18]. The annotation of gene functions and metabolic pathways based on existing databases, containing predictions such as Motif, structural domains, protein activities, and information about the metabolic pathways in which they are placed, is referred to as functional annotation of genes. Gene function annotation was performed on strain MNP-2 using nine databases, including Nr (https://ftp.ncbi.nlm.nih.gov), Pfam (https://pfam.xfam.org/), eggCOG (https://www.ncbi.nlm.nih.gov/COG/), Uniprot (https://www.uniprot.org/), KEGG (https://www.kegg.jp/kegg/), GO (http://geneontology.org/), Pathway (http://www.pathwaycommons.org/), Refseq (https://www.ncbi.nlm.nih.gov/refseq/), Interproscan (https://github.com/ebi-pf-team /interproscan), and so on, in order to acquire comprehensive gene function information.

### Non-coding RNA annotation

Non-coding RNAs can all be transcribed from the genome, but rather than being translated into proteins, they can carry out their biological tasks at the RNA level. TRNA and rRNA are two of them that are directly engaged in protein synthesis. Using INFERNAL (v1.1.2, https://github.com/EddyRivasLab/in fernal) software based on the Rfam database (http://rfam.xfam.org/), various forms of ncRNAs were predicted and statistically categorised.

### Repetitive sequence annotation

Scattered repeats and tandem repeats are two types of repeated sequences. LTR, LINE, SINE, and DNA transposons are examples of scattered repetitive sequences, also known as transposon elements. They can be characterized as highly repetitive sequences, moderately repetitive sequences, or low repetitive sequences based on the number of repeats. The software RepeatModeler (v1.0.4, https://github.com/Dfam consortiu m/RepeatModeler) was used to create its own repeat library, and RepeatMasker (v4.0.5, https://github.com/rmhubley/RepeatMasker) was used to annotate the genome with repetitive sequences.

### Prediction of carbohydrate-active enzymes (CAZymes)

CAZymes are a very important class of enzymes classified as Glycoside Hydrolases (GHs), Glycosyl Transferases (GTs), Polysaccharide Lyases (PLs), Carbohydrate Esterases (CEs), Auxiliary Activities (AAs), Carbohydrate-Binding Modules (CBMs), and so on. The research of carbohydrate-related enzymes can yield a lot of useful biological information. The CAZy database can be used to investigate carbohydrase genomic, structural, and biochemical information. HMMER (v3.2.1, https://github.com/EddyRivasLab/hmmer) was used to annotate protein sequences based on the CAZy database (filtering parameters: E-value < e^− 18^, coverage > 0.35, http://www.cazy.org/).

### Analysis of pathogen-host interaction (PHI)

PHI is a database of pathogen-host interactions with experimentally validated content derived primarily from fungal, oomycete, and bacterial pathogen-infected hosts such as animals, plants, and insects. The target protein sequences were annotated using Diamond blastp (v2.9.0, https://github.com/enormandeau/ ncbi_b last_tutorial) based on the PHI database (http://www.phi-base.org).

### Prediction of drug-resistant gene

The CARD framework is built as an Antibiotic Resistance Ontology (ARO) taxonomic unit to correlate information on antibiotic modules and their targets, resistance mechanisms, gene variants, etc. The comparison results display the position of each gene annotated in the CARD database (https://card.mc maste r.ca/), as well as the ARO ID and classification description, which can be used to understand the specific function of each gene related to antibiotic resistance.

### Cytochromes P450 (CYP450) annotation

CYP450 is a large protein family that catalyzes the oxidation of a variety of substrates and participates in the metabolism of endogenous and exogenous substances such as drugs and environmental compounds. The target protein sequences were annotated using Diamond blastp based on the FungalP450 database (http://drnelson.utmem.edu/CytochromeP450.html).

### Prediction of virulence gene

Database of fungal virulence factors (DFVF, http://sysbio.unl.edu/DFVF/) is a database dedicated to the study of fungal virulence factors. To investigate the virulence-related genes present in strain MNP-2, the predicted protein sequences were compared with DFVF using Diamond blastp.

### Other annotations

Classification of membrane transporter proteins using Transporter Classification Database (TCDB, http://www.tcdb.org/). All predicted gene pair protein sequences were analyzed using the software signalP (v5.0, http://www.cbs.dtu.dk/services/SignalP/) to identify proteins containing signal peptides. To identify proteins containing transmembrane helices and secreted proteins, all predicted gene-to-protein sequences were analyzed using the software tmhmm (v2.0, http://www.cbs.dtu.dk/services/TMHMM/).

### Prediction of biosynthetic gene clusters (BGCs)

The genes responsible for secondary metabolite production are typically organized in BGCs. antiSMASH (v7.1.0, https://docs.antismash.secondarymetabolites.org/) is the most extensively used tool for finding and characterizing BGCs in bacteria and fungi at the moment. antiSMASH employs a rule-based technique to detect a variety of SM-producing biosynthetic pathways. For BGCs encoding NRPSs, type I and type II PKSs, lanthipeptides, lasso peptides, sactipeptides, and thiopeptides, which cluster-specific analyses can provide more information about the biosynthetic steps performed and thus provide more detailed predictions on the compounds produced, more in-depth analyses are performed.

### Analysis of molecular networking (MN)

MN has swiftly become an extensively used technology in the field of natural products chemistry, with applications ranging from dereplication to genome mining, metabolomics, and chemical space visualization since the advent of the online open-source Global Natural Products Social (GNPS, https://gnps.ucsd.edu/). The samples were dissolved in methanol (1 mg/mL) and analyzed using a SCIEX X500 QTOF (SCIEX, USA) mass spectrometer to generate LC-MS/MS data, which were pre-processed by Mzmine and analyzed on the GNPS online platform to generate molecular networks, which were visualized using Cytoscape. Separation was done on a chromatographic column (Phenomenex, H19-046789, 1.7 μm C18 100 Å, 150 × 2.1 mm, maintained at 40 °C with a flow rate of 0.3 mL/min). A linear gradient system composed water and acetonitrile was used, starting from 10% (vol/vol) acetonitrile and increasing to 100% in 20 min. Samples were analyzed in positive electrospray scanning, m/z 50 − 1,500.

### Fermentation and extraction

The strain MNP-2 grown on potato dextrose agar (PDA) medium was inoculated into 500 mL Erlenmeyer flasks containing 200 mL potato dextrose broth (PDB) medium, and shaken for 3 days at 200 rpm and 30 ℃. The fermentation was performed in Erlenmeyer flasks (2 × 1 L) with sterilized rice (80 g) and tap water (120 mL). After autoclaving at 121 ℃ for 20 min, each flask was inoculated with 5% seed cultures and then incubated at room temperature under static conditions for 30 days. The fermented rice in each flask was extracted with 500 mL EtOAc by an ultrasonic instrument for 20 min three times followed by filtration using gauze. All the filtrate was combined and evaporated under vacuum to dryness, obtaining the sample 1 (approx. 1.56 g). The strain MNP-2 was inoculated in 500 mL flasks containing 200 mL Czapek-Dox or PDB Medium (2 flasks each) and shaken for 15 days at 200 rpm and 30 ℃. After completion of fermentation, the fermentation broth was extracted three times by EtOAc (twice the volume of the fermentation broth) and evaporated under vacuum to dryness, obtaining samples 2 (approx. 0.27 g) and 3 (approx 0.31 g). The culture medium composition is shown in the supporting material.

### Antimicrobial assay

Antimicrobial activities were carried out according to the filter paper disc (5 mm in diameter) diffusion technique [19, 20]. Two human pathogenic strains of *Staphylococus aureus* ATCC 25,923 and *Escherichia coli* ATCC 25,922 were obtained from Nanjing Medical University (China). The pathogenic bacteria were incubated in Luria-Bertani (LB) medium at 37 °C for 24 h and then spread evenly on LB solid plates. The samples were dissolved in dimethyl sulfoxide (DMSO) at a concentration of 10 mg/mL for crude extract and 2 mg/mL for ampicillin sodium. Each disc was impregnated with 20 µl of samples and ampicillin sodium. Discs with DMSO (20 µl) served as a control.

The discs were dried at 37 °C for 2 h and introduced to the surface of the medium (Containing the pathogenic bacteria) using sterile tweezers. The plates were incubated at 37 °C for 24 h to obtain zones of inhibition. The experiments were repeated three times. 

## Results

### Morphology, classification and phylogenetic analysis of strain MNP-2

**Fig. 1:**
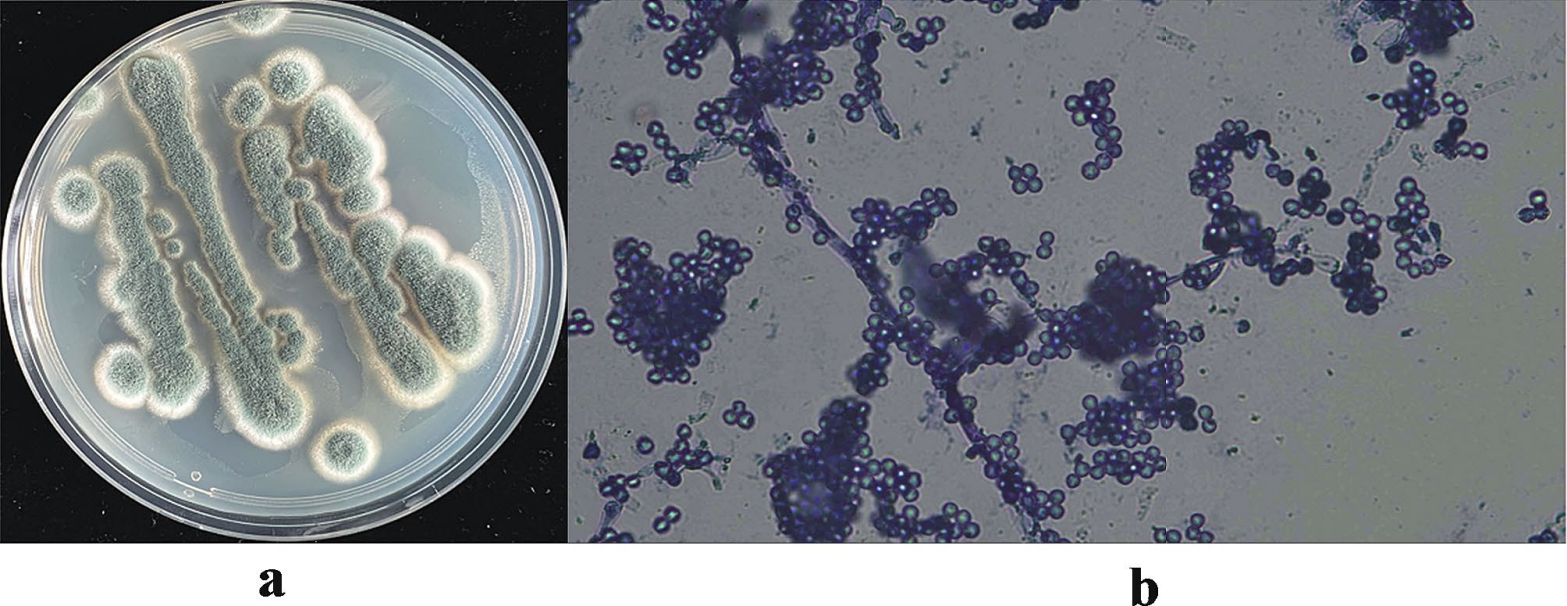
Morphology analysis of strain MNP-2

On potato dextrose agar PDA medium, strain MNP-2 grows quickly, starting out as white filamentous, turning green in 2–3 days, and eventually turning dark green and powdery in a few days (Fig. 1a). Mycelium is more branched, septate multinucleate, conidial peduncle apical expansion into a spherical apical capsule, and a small peduncle bearing a string of conidia, according to electron microscope observations (Fig. 1b).

**Fig. 2:**
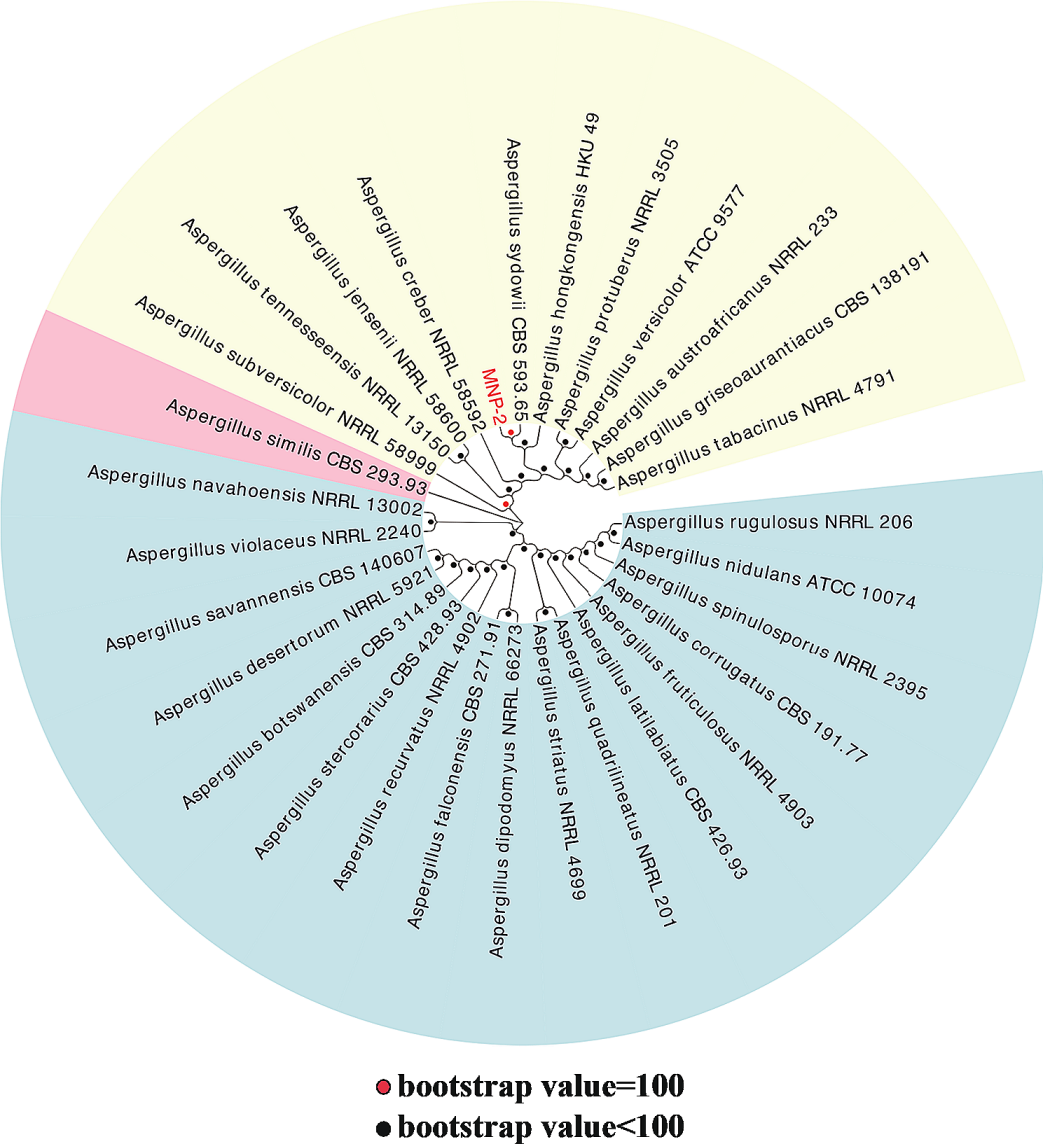
The ITS gene sequences-based phylogenetic tree of strain MNP-2. All strain from the NCBI rRNA/ITS database

The nuclear ribosomal DNA (nr DNA) internal transcribed spacer region (ITS) of strain MNP-2 was amplified and sequenced, and the ITS sequence was searched for homology in the nucleic acid database genbank. Blast analysis [21] of the ITS gene sequence revealed that strain MNP-2 had the highest similarity with the strain *A. sydowii* CBS 593.65 (100%, Fig. 2).

### Genome feature of strain MNP-2

**Fig. 3:**
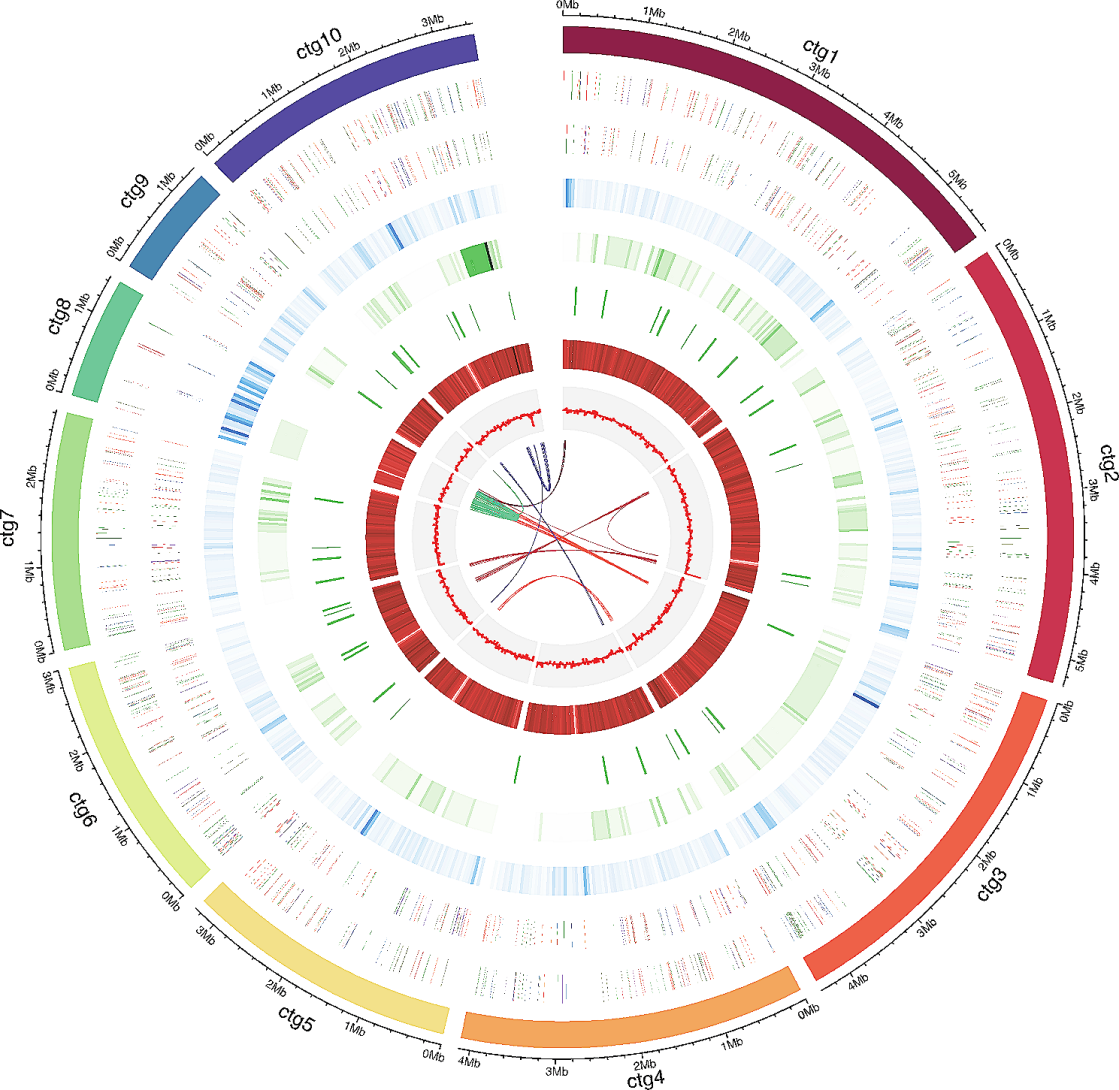
Genome diagram of strain MNP-2. From inside to outside, the first line shows in-paralog pairs; The second circle shows GC skew; The third circle shows GC content; The fourth circle shows putative BGCs; The fifth circle shows nc RNA; The sixth shows repeat; The seventh circle and the eighth circle display protein-encoding sequences (CDSs) annotation information, and different colors represent different COG function classification. The seventh circle indicates that CDSs is in a negative chain, and the eighth circle indicates that CDSs is in a positive chain. The outer rim shows the scaffold

The whole genome of strain MNP-2 contains 10 contigs with an N50 of 4.1 Mb and 50.0% GC content (Table 1), and its size was determined as 34.9 Mb (Fig. 3). N50 is the shortest contig length that needs to be included for covering 50% of the genome. In general, the contig N50 size of the genome is used to assess genome continuity; the larger the contig N50, the better the genome continuity. The overall genomic characteristics are similar to those of the five strains of *A. sydowii* currently included in the NCBI database (https://www.ncbi.nlm.nih.gov/) (Fig. S2).

**Table 1:** Statistics of strain assembly

Item	Value
Total_length (bp)	34,924,093
Total_length_without N (bp)	34,924,093
Contig	10
GC_content (%)	50.00
N50 (bp)	4,126,853
N90 (bp)	2,836,306
Average (bp)	3,492,409.30
Median (bp)	3,372,540.50
Min (bp)	1,368,430
Max (bp)	5,687,656

^1^ Total_length is the assembly length; Total_length_without N is the length without gap in the assembly result; GC_content is the GC content; N50 is the N50 of the contigs. When the added length reaches half of the total length, the length of the last added contig is N50; the N90 algorithm is the same as N50; Average is the average length of the contig; Median is the median length of the contig; Min is the minimum length of the contig; Max is the maximum contig length. Generally, the contig N50 size of the genome is used to evaluate the continuity of the genome

### Prediction of genetic structure

The prediction and completeness evaluation of coding genes showed that there were 13,218 total genes, with an average mRNA length of 1,610.07 bp, an average CDS length of 1,444.90 bp, a total of 42,982 exons, a total of 29,764 introns, an average number of exons per gene of 3.25, an average exon length of 444.34 bp, and an average intron length of 29,764 bp. The gene had an average exon count of 3.25, an average exon length of 444.34, an average intron length of 73.35, and a single copy BUSCO of 98% [22] (Table S1, Fig. S3). Results of non-coding RNA annotation for rRNA, sRNA, snRNA, and tRNA were 46, 7, 32, and 119, respectively (Table S2). According to the repeat sequence annotation results, there were 19 SINE (Short interspersed element), 414 LINE (Long interspersed nuclear element), 1,728 L (Long terminal repeat), 606 DNA transposons, 40 satellite DNAs, and 91 others (Table S3).

### Annotation of gene functions

**Fig. 4:**
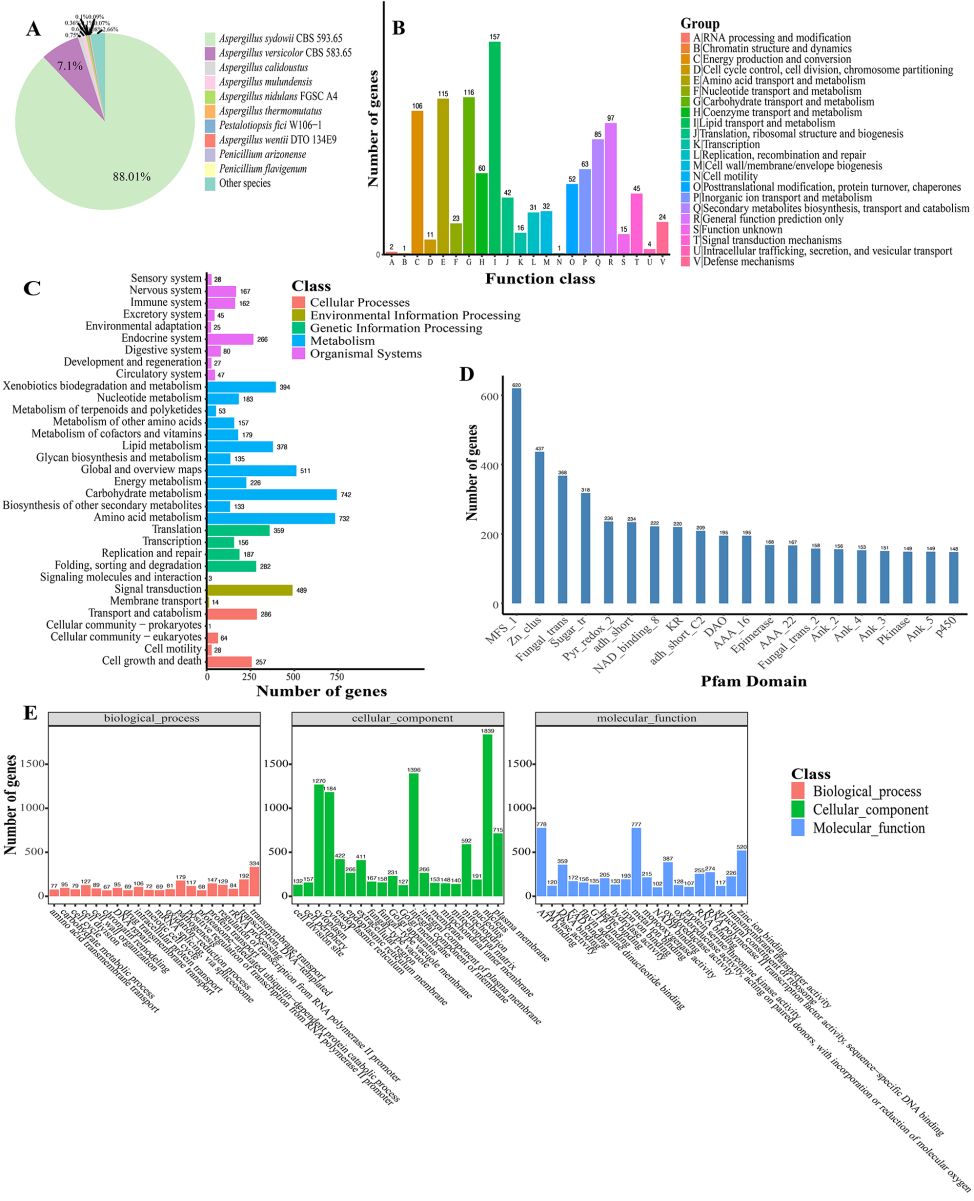
Annotation of gene function. **(a)** Distribution of species obtained from Non-redundant protein sequence (Nr) database matching. **(b)** Classification statistics with Cluster of Orthologous Group of proteins (COG) annotations. **(c)** Functional classification of Kyoto Encyclopedia of Gene and Genome (KEGG) pathway. **(d)** Classification statistics with Pfam annotations. **(e)** Classification statistics with Gene Ontology (CO) annotations

The total number of predicted genes was 13,218 of these, the number of genes with annotation information was 12,912 (97.68%), and the total number of functional gene annotations in the databases of Nr, Pfam [23], eggCOG [24], Uniprot [25], KEGG [26], GO [27], Pathway [28], Refseq [29], and Interproscan [30] were 12,894 (97.55%), 10,642 (80.39%), 1,047 (7.92%), 7,772 (58.8%), 2,946 (22.29%), 7,693 (58.20%), 2,764 (20.91%), 6,252 (47.30%), 10,626 (80.39%, Table S4), respectively. According to the analysis of the Nr library comparison annotation results, the species with the most strain MNP-2 comparisons was *Aspergillus* sp. fungi (Fig. 4a). The COG database, which was created based on the evolutionary connections between bacteria, algae, and eukaryotes, can be used to categorize genes according to their direct homology. Energy generation and conversion, amino acid transport and metabolism, carbohydrate transport and metabolism, and lipid transport and metabolism are the COG group’s more prevalent categories, according to the examination of COG data (Fig. 4b). The sequences’ major classification after KEGG annotation was broken down into cellular processes, environmental information processing, genetic information processing, metabolism, cellular systems, etc. Among them, metabolism (3,823, 56.25%) has the most annotated genes, particularly involved in carbohydrate metabolism and amino acid metabolism, with 742 and 732 annotated genes, respectively. These annotated genes suggest the existence of rich and diverse functions for protein and lipid metabolism, resulting in higher energy conversion efficiency (Fig. 4c). The GOslim classification was obtained by simplifying the GO annotation information, and the top 20 most annotated GOslim secondary classifications under each classification were chosen for mapping (Fig. 4e) after summarizing the gene functions in terms of cellular components, molecular functions, and biological processes. The gene enrichment of each GO secondary function in the context of all genes was used to understand the status of each secondary function. The Pfam database contains information about protein families. The genes annotated in each structural domain were statistically summarized, and the top 20 annotated structural domains were mapped (Fig. 4d), with the number of genes matching on the Major Facilitator Superfamily (MFS) found to be the highest, 620, according to the annotations.

### Annotations to proprietary databases

In addition to the studies mentioned above, 6 carbohydrate-related enzymes were annotated using the Carbohydrate-active enzymes (CAZy) database [31](Table S5). Based on the Pathogen Host Interactions (PHI) database [32], 6 assay sequences were annotated, and the target sequences and similarity of the database matches were given (Table S6). 10 drug resistance genes were annotated using the Comprehensive Antibiotic Research database (CARD) [33]to learn more about the drug resistance genes present in each genome (Table S7). On the basis of the FungalP450 database [34], a total of 1366 cytochrome P450 (CYP450) protein sequences were annotated. CYP450 is a broad family of proteins with ferroheme as a cofactor (Table S8). The predicted protein sequences were compared with the Database of Fungal Virulence Factors (DFVF) [35], and a total of 6 virulence-related genes were found in the sequenced strains (Table S9). Meanwhile, 2139 membrane transport proteins were annotated in the Transporter Classification Database (TCDB) [36], 1182 protein sequences containing signal peptides were predicted by SignalP software, and 2729 protein sequences containing transmembrane proteins and 926 protein sequences containing secreted proteins were predicted by TMHMM (Table S10). The analysis software and database information used in the study are shown in Table S11.

#### Prediction of secondary metabolite clusters (BGCs)

**Fig. 5:**
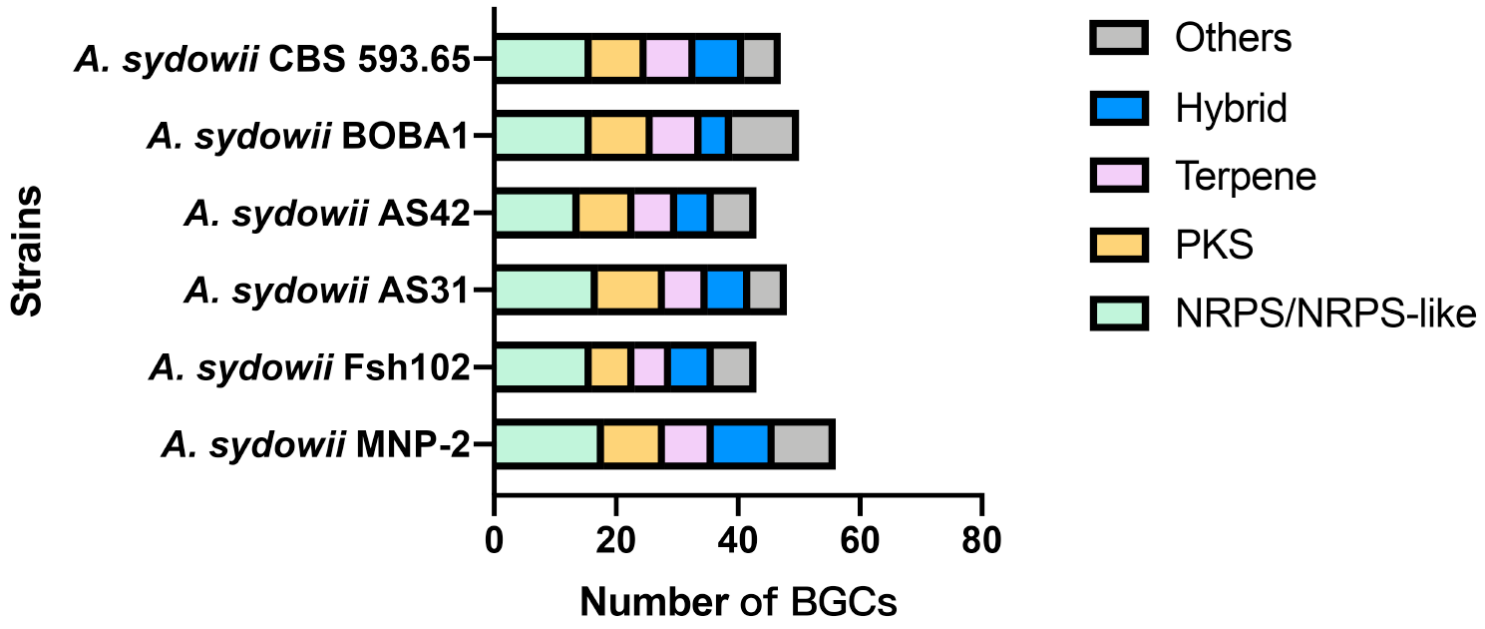
BGC analysis of *A. sydowii*

**Fig. 6:**
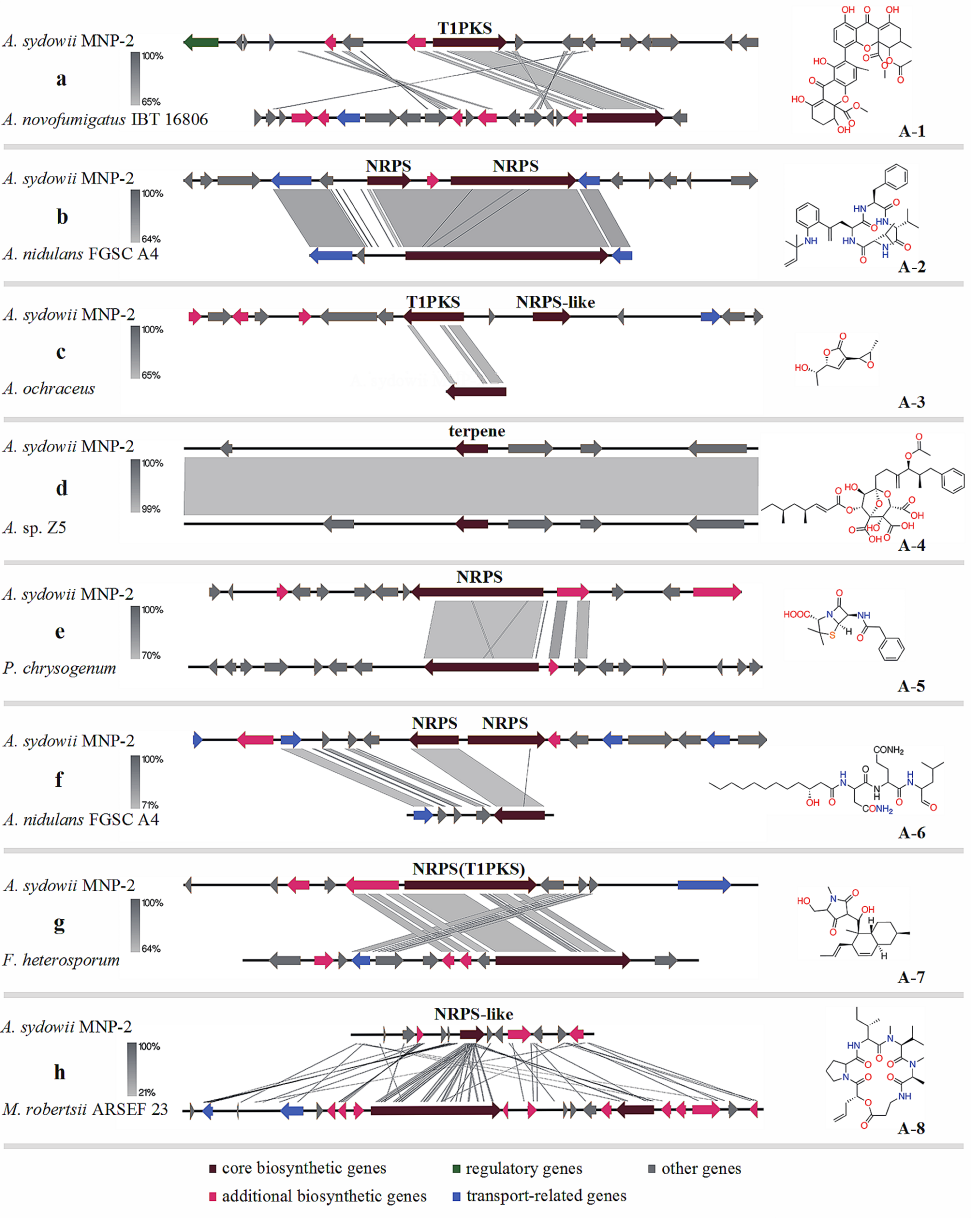
Synteny plots of investigated clusters made using Easyfig BLASTn. **(a)** The synteny of the predicted neosartorin A cluster in strain MNP-2 (Location 1,037,103-1,079,796 nt) and the identified candidate cluster in *A. novofumigatus* IBT 16,806. **(b)** The synteny of the predicted nidulanin A cluster in strain MNP-2 (Location 4,611,786-4,665,953 nt) and the identified candidate cluster in *A. nidulans* FGSC A4. **(c)** The synteny of the predicted asperlactone cluster in strain MNP-2 (Location 449,413–499,803 nt) and the identified candidate cluster in *Aspergillus ochraceus*. **(d)** The synteny of the predicted squalestatin cluster in strain MNP-2 (Location 3,433,532-3,454,746 nt) and the identified candidate cluster in *Aspergillus* sp. Z5. **(e)** The synteny of the predicted penicillin cluster in strain MNP-2 (Location 219,956 − 272,605 nt) and the identified candidate cluster in *Penicillium chrysogenum.***(f)** The synteny of the predicted fellutamide B cluster in strain MNP-2 (Location 220,650 − 272,412 nt) and the identified candidate cluster in *A. nidulans* FGSC A4. **(g)** The synteny of the predicted equisetin cluster in strain MNP-2 (Location 777,609–829,611 nt) and the identified candidate cluster in *Fusarium heterosporum.***(h)** The synteny of the predicted destruxin A cluster in strain MNP-2 (Location 2,262,285-2,306,801 nt) and the identified candidate cluster in *Metarhizium robertsii* ARSEF 23. The core biosynthetic genes has been highlighted with red, the regulatory genes has been highlighted in green, the additional biosynthetic genes has been highlighted in pink, the transport-related genes has been highlighted in blue and and gray markers represent genes without functional annotation

SM clusters prediction in *A. sydowii* was done using an available software packages antiSMASH [37]. A total of 287 putative BGCs were detected in the 6 genomes, corresponding to an average of 47.8 per strain, with the highest number of 56 BGCs being observed in strain MNP-2 (*A. sydowii* Fsh102 43 SM clusters, *A. sydowii* AS31 48 SM clusters, *A. sydowii* AS42 43 SM clusters, *A. sydowii* BOBA1 50 SM clusters, *A. sydowii* CBS 593.65 47 SM clusters, Fig. 5). The predicted SM clusters of strains are defined by the “backbone enzymes” that generate the putative SM’s carbon skeleton. The majority of the “backbone enzymes” in strain MNP-2 are polyketide synthase (PKS) or non-ribosomal peptides synthase (NRPS). 18 SM clusters contain sequence coding for a NRPS/NRPS-like enzymes, 10 SM clusters contain sequence coding for a PKS/PKS-like enzymes and 10 SM clusters are hybrid clusters. The remaining SM clusters seem to be required in terpene/terpenoid metabolites production as the “backbone enzyme” is a terpene cyclase (8 SM clusters) or indole synthase (5 SM clusters). These predicted BGCs reveal that strain MNP-2 has a diverse variety of secondary metabolite production potential, and some of them are strikingly similar to BGCs from compounds like neosartorin (A-1) [38], nidulanin A (A-2) [39], asperlactone (A-3) [40], squalestatin (A-4) [41], penicillin (A-5) [42], fellutamide B (A-6) [43], equisetin (A-7) [44], destruxin A (A-8) [45], and others that have been reported in current publications (Fig. 6). These compounds are all secondary metabolites produced by microorgnisms and exhibit a variety of biological functions (Table S12). Some of the substances mentioned above demonstrate the ability of strain MNP-2 to produce these kinds of substances.

#### Analysis of molecular networking (MN)

**Fig. 7:**
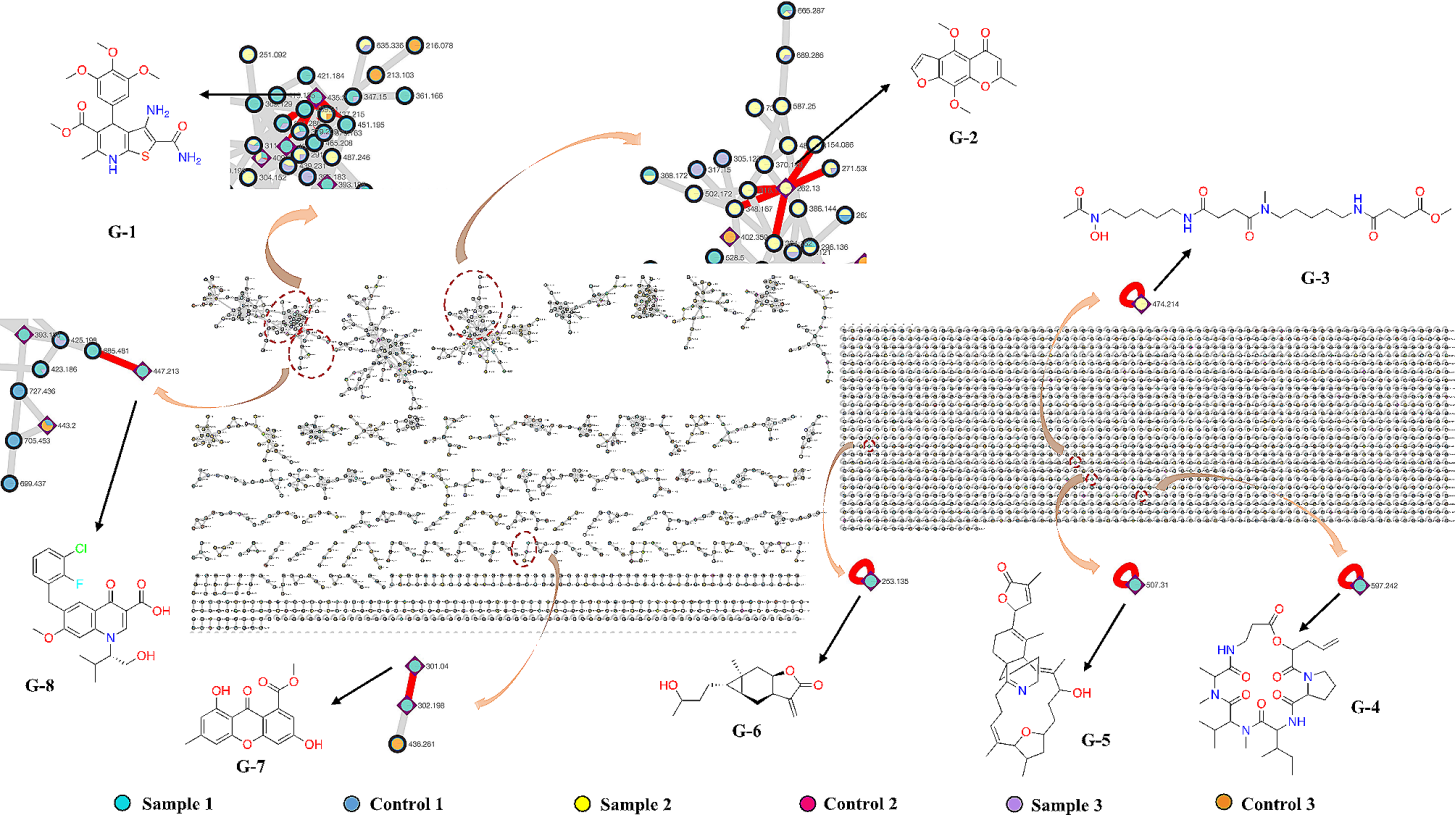
Molecular network analysis of strain MNP-2-derived secondary metabolites. Sample 1 is the crude extract of strain MNP-2 grown on rice-solid medium for 30 days (cyan node), with the sterile rice-solid medium crude extract serving as control 1(blue node); Sample 2 is the crude extract of strain MNP-2 cultivated in Czapek-Dox medium for 15 days (yellow node), with the sterile Czapek-Dox medium crude extract serving as control 2 (red node); Sample 3 is the crude extract of strain MNP-2 cultivated PDB for 15 days (purple node), with the sterile PDB medium extract serving as control 3 (orange node)

The metabolite analysis demonstrated that strain MNP-2 has the capacity to create complex molecular skeletons, particularly some heterocyclic or compound skeletons with a bridge-ring (G-1 - G-8, Fig. 7). These compounds come from a wide range of sources and have diverse biological activities (Table S13) [46–50]. Destruxin A (A-8), a cyclic peptide with strong bioactivity, was also found in sample 1 (Fig. 7, G-4), and the antiSMASH analytical platform was used to look into its potential BGC (Fig. 6h). Similarly, neosartorin (A-1), a xanthone analogue, and asperlactone (A-3), which contains a lactone ring, were both found to have similar structures present in the MN (G-6, G-7), greatly facilitating the analysis of biosynthetic pathways for this class of compounds. Furthemore, the majority of the metabolites analyzed were not matched to the corresponding BGCs, indicating a significant research gap that needs to be filled.

The analysis revealed that the metabolites of strain MNP-2 were extremely abundant, and their structural features mainly included aromatic polyketides, peptides, alkaloids, terpenoids, and fatty acids. In rice-solid medium, strain MNP-2 had the most abundant metabolites, as shown in Fig. 7. Furthermore, there are more monochromatic block nodes in the molecular network, indicating that strain MNP-2 has significant metabolic differences in different mediums. There were many unknown nodes present in clusters, and some of the marker compounds were closely related to the BGCs of strain MNP-2.

#### Antimicrobial activity

The antibacterial activity of these samples was evaluated by the filter paper disc diffusion technique, with ampicillin sodium as the positive control (Fig. S1). The results showed that samples 1 and 2 had moderate inhibitory effects on S. aureus ATCC 25923, with inhibition zones of 10.04 ± 0.49 and 10.40 ± 0.36 mm, respectively.

## Discussion

Till now, only five complete genome sequences of *A. sydowii* strains from various sources had been deposited in the NCBI database. Comparative analysis of the total gene features of these strains with those of *A. sydowii* MNP-2 showed that the strain MNP-2 possesses the middle level of genome size (34.9 Mb) and GC content (50%). However, the contig number of strain MNP-2 was only 10, suggesting the quality of genome assembly is more higher than others.

AntiSMASH study revealed that strain MNP-2 possessed 56 BGCs, including NRPS/NRPS-like (18 SM clusters), PKS/PKS-like (10 SM clusters), terpene cyclase (8 SM clusters), indole synthase (5 SM clusters), heterozygous route (10 SM clusters), and fungal-RiPP (5 SM clusters). Compared to the strains *A. sydowii* Fsh102, *A. sydowii* AS31, *A. sydowii* AS42, and *A. sydowii* BOBA1, *A. sydowii* CBS 593.65, the strain *A. sydowii* MNP-2 had the highest number of BGCs. Some BGCs may manufacture several complicated structurally active compounds (such as A-1 - A-8) [38–45]. However, the majority of the other BGCs do not match similar clusters that might synthesize unique chemical skeleton. Although the types of BGCs can be accurately predicted based on the core SM biosynthetic genes encoding backbone enzymes, it is still impossible to exactly predict the boundaries of BGCs or the functions of some clusters without backbone enzymes. This is due to the fact that a large number of genes around the core SM biosynthetic genes in strain MNP-2 cannot been characterized using open-source bioinformatics tools [51, 52]. Metabolic analyses of strain MNP-2 grown on various media (rice-solid, Czapek-Dox and PDB) using GNPS networking revealed its great potential of biosynthesis of bioactive SMs containing a variety of heterocyclic and bridge-ring structures. For example, compound G-8 exhibited potent anti-HIV effect with an IC_50_ value of 7.2 nM and an EC_50_ value of 0.9 nM [50]. Compound G-6 had excellent in vitro cytotoxicities against the K562, MCF-7, Hela, DU145, U1975, SGC-7901, A549, MOLT-4 and HL60 cell lines with IC_50_ values ranged from 0.10 to 3.3 µM, and showed significant anti-viral (H1N1 and H3N2) activities with IC_50_ values of 15.9 and 30.0 µM, respectively [48]. These findings indicate that the Arctic marine-derived strain MNP-2 is one of prolific producers of therapeutic agents. To deeply mine the biosynthetic potential of strain MNP-2, leveraging genomic and metabolomic data rapidly facilitates assessing the novelty of metabolites and linking them to their BGCs [53–55].

## Conclusions

As one of underexploited organisms on earth, polar marine-derived microbes harbor more diversified genes for the biosynthesis of functional natural products1. In this study, a high-quality whole genome sequence of an Arctic marine strain MNP-2 with a size of 34.9 Mb was successfully obtained. Its total number of genes predicted by BRAKER software was 13,218, and that of non-coding RNAs (rRNA, sRNA, snRNA, tRNA) predicted by using INFERNAL software was 204. The number of annotated genes was found to be 12,912, accounting for 97.68% of all genes using the Nr, Pfam, eggCOG, KEGG and GO databases. The results of these analyses are significant for gene resource mining and polar microbial genome investigations. Additionally, antiSMASH results indicated that strain MNP-2 harbors 56 BGCs, which can produce SMs with various structure motifs. This work effectively unveiled the biosynthetic potential of strain MNP-2 using genomics and metabolomics techniques. Various genome mining strategies should be further employed to awaken most cryptic BGCs in this strain to produce novel and/or valuable SMs [56], such as ribosome engineering [57], metabolic engineering [58], global regulators [59], protein modification genes [60], heterologous expression [61], promoter exchange [62], BGC refactoring [63], BGC-specific regulators [64].

### Electronic supplementary material

Below is the link to the electronic supplementary material.

Supplementary Material 1

## Data Availability

The complete genome sequence data reported in this study are available within NCBI GCA_034192605.1.

## Abbreviations

SMs: secondary metabolites
MN: molecular networking
GNPS: Global Natural Products Social Molecular Networking
BGCs: biosynthetic gene clusters
PDA: potato dextrose agar
PDB: potato dextrose broth
PBS: phosphate buffered solution
CAZymes: carbohydrate-active enzymes
GHs: Glycoside Hydrolases
GTs: Glycosyl Transferases
PLs: Polysaccharide Lyases
CEs: Carbohydrate Esterases
AAs: Auxiliary Activities
CBMs: Carbohydrate-Binding Modules
PHI: pathogen-host interaction
ARO: Antibiotic Resistance Ontology
CYP450: Cytochromes P450
DFVF: Database of fungal virulence factors
TCDB: Transporter Classification Database
ITS: internal transcribed spacer region
CDSs: protein-encoding sequences
SINE: Short interspersed element
LINE: Long interspersed nuclear element
LTR: Long terminal repeat
MFS: Major Facilitator Superfamily
Nr: Non-redundant protein sequence
COG: Cluster of Orthologous Group of proteins
KEGG: Kyoto Encyclopedia of Gene and Genome
CO: Gene Ontology
CARD: Comprehensive Antibiotic Research database
TCDB: Transporter Classification Database
PKS: polyketide synthase
NRPS: non-ribosomal peptides synthase
