# Supplementary material for: Unveiling biosynthetic potential of an Arctic marine-derived strain Aspergillus sydowii MNP-2
Source: BMC Genomics. 2024 Jun 17;25:603. doi: 10.1186/s12864-024-10501-0 (PMC11181645; doi:10.1186/s12864-024-10501-0)
Supplement: Supplementary file 1 — Supplementary Material 1 [file 12864_2024_10501_MOESM1_ESM.docx]

**Supplementary Information for**

Unveiling biosynthetic potential of an Arctic marine-derived strain *Aspergillus sydowii* MNP-2

**Zhiyang Fu, Xiangzhou Gong, Zhe Hu, Bin Wei, Huawei Zhang ***

***** Correspondence: [hwzhang@zjut.edu.cn](mailto:hwzhang@zjut.edu.cn); Tel.: 86-571-88320913

School of Pharmaceutical Sciences, Zhejiang University of Technology, Hangzhou, 310014, China

**Content**

[Supplementary Fig. 1. Evaluation of antimicrobial activity of crude extract. 2](#_Toc18297)

[Supplementary Fig. 2. Maximum likelihood phylogram and genome statistics of *A. sydowii* species analysed in this study. 3](#_Toc12590)

[Supplementary Table 1. Statistics of annotation results of coding genes. 4](#_Toc31662)

[Supplementary Fig. 3. Results of BUSCO evaluation of coding genes. 5](#_Toc21382)

[Supplementary Table 2. Statistics of annotation results of non-coding RNA 6](#_Toc31592)

[Supplementary Table 3. Statistics of annotation results of repeat sequence. 7](#_Toc13614)

[Supplementary Table 4. Statistics of annotation results. 8](#_Toc29835)

[Supplementary Table 5. Statistics of annotation results of CAZy database. 9](#_Toc11674)

[Supplementary Table 6. Statistics of annotation results of PHI database. 10](#_Toc9576)

[Supplementary Table 7. Statistics of annotation results of CARD database. 11](#_Toc31681)

[Supplementary Table 8. Statistics of annotation results of CYP450. 12](#_Toc2911)

[Supplementary Table 9. Statistics of annotation results of DFVF database. 13](#_Toc185)

[Supplementary Table 10. Statistics of annotation results of TCDB database, SignalP and tmhmm software. 14](#_Toc24445)

[Supplementary Table 11. Statistics on Software and Database. 15](#_Toc17509)

[Supplementary Table 12. antiSMASH and MN analyze information about SMs. 16](#_Toc6399)

[ITS gene sequence 17](#_Toc15470)

[Culture medium 17](#_Toc11483)

[References 18](#_Toc20503)

Supplementary Fig. 1. Evaluation of antimicrobial activity of crude extract.


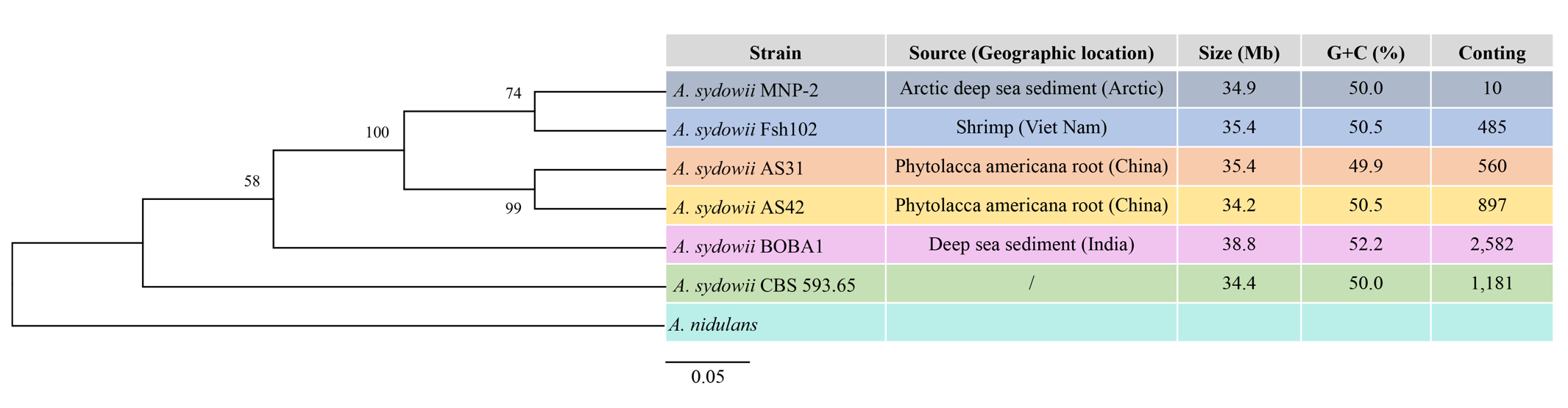


Supplementary Fig. 2. Maximum likelihood phylogram and genome statistics of *A. sydowii* species analysed in this study. *A. sydowii* MNP-2 (GCA_034192605.1), *A. sydowii* Fsh102 (GCA_009193685.1), *A. sydowii* AS31 (GCA_026319385.1), *A. sydowii* AS42 (GCA_026319405.1), *A. sydowii* BOBA1 (GCA_009828905.1), *A. sydowii* (GCA_001890705.1), *A. nidulans* (GCF_000011425.1). Genome phylogenetic analysis by UFCG database [1].

Supplementary Table 1. Statistics of annotation results of coding genes.

| **Type** | **Number** |
| --- | --- |
| The total number of gene | 13,218 |
| The average of mRNA_length | 1,610.07 |
| The average of cds_length | 1,444.90 |
| The average of exon_number | 3.25 |
| The average of exon_length | 444.34 |
| The average of intron_length | 73.35 |
| The total number of exon | 42,982 |
| The total number of intron | 29,764 |
| The total intron length | 2,183,306 |


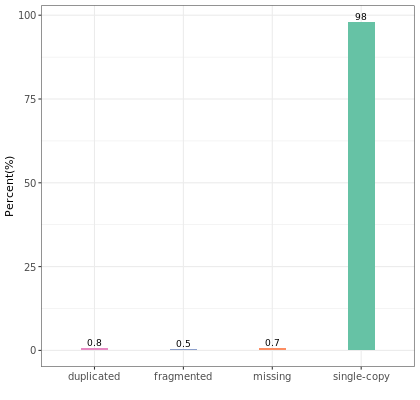


Supplementary Fig. 3. Results of BUSCO evaluation of coding genes.

Supplementary Table 2. Statistics of annotation results of non-coding RNA

| **Class** | **Number** | **Total length (bp)** | **Mean length (bp)** |
| --- | --- | --- | --- |
| rRNA | 46 | 36,443 | 792 |
| sRNA | 7 | 1,699 | 242 |
| snRNA | 32 | 3,960 | 123 |
| tRNA | 119 | 10,310 | 86 |

Supplementary Table 3. Statistics of annotation results of repeat sequence.

| **Item** | **Number** | **Length (bp)** | **Coverage (%)** |
| --- | --- | --- | --- |
| SINE | 19 | 1,642 | 0.00 |
| LINE | 414 | 105,636 | 0.30 |
| LTR | 1,728 | 222,650 | 0.64 |
| DNA | 606 | 153,022 | 0.44 |
| Satellite | 40 | 3,931 | 0.01 |
| Simple_repeat | 5,064 | 195,185 | 0.56 |
| Low_complexity | 813 | 39,401 | 0.11 |
| Other | 67 | 5,503 | 0.02 |
| Unknow | 24 | 2,730 | 0.01 |
| Total | 8,003 | 619,922 | 1.78 |

Supplementary Table 4. Statistics of annotation results.

| **Item** | **Count** | **Percentage (%)** |
| --- | --- | --- |
| All | 13,218 | 100 |
| Annotation | 12,912 | 97.68 |
| Uniprot | 7,772 | 58.80 |
| Pfam | 10,642 | 80.51 |
| Refseq | 6,252 | 47.30 |
| Nr | 12,894 | 97.55 |
| Interproscan | 10,626 | 80.39 |
| GO | 7,693 | 58.20 |
| KEGG | 2,946 | 22.29 |
| Pathway | 2,764 | 20.91 |
| COG | 1,047 | 7.92 |

Supplementary Table 5. Statistics of annotation results of CAZy database.

| **Family HMM** | **HMM**  **length** | **Query ID** | **Query**  **length** | **E-value** | **HMM**  **start** | **HMM**  **end** | **Query**  **start** | **Query**  **end** | **Coverage(%)** |
| --- | --- | --- | --- | --- | --- | --- | --- | --- | --- |
| GH28.hmm | 325 | g10004.t1 | 399 | 1.9e^-70^ | 6 | 308 | 71 | 388 | 92.92 |
| GH3.hmm | 216 | g10008.t1 | 1,035 | 5.1e^-60^ | 4 | 216 | 220 | 435 | 98.15 |
| GH28.hmm | 325 | g10026.t1 | 433 | 2.1e^-74^ | 9 | 323 | 71 | 424 | 96.62 |
| GH32.hmm | 293 | g10059.t1 | 641 | 4.8e^-68^ | 1 | 284 | 50 | 409 | 96.59 |
| GH13_1.hmm | 306 | g10060.t1 | 554 | 4.1e^-121^ | 17 | 306 | 1 | 290 | 94.44 |
| CBM20.hmm | 90 | g10060.t1 | 554 | 8e^-31^ | 2 | 88 | 454 | 543 | 95.56 |

Supplementary Table 6. Statistics of annotation results of PHI database.

| **Qseqid** | **Sseqid** | **Pident (%)** | **length** | **Mismatch** | **Gapopen** |
| --- | --- | --- | --- | --- | --- |
| G5188.t1 | T2C913 | 43.649 | 433 | 217 | 7 |
| G5188.t1 | A0A0J9WXI0 | 27.451 | 204 | 142 | 4 |
| G5190.t1 | Q32WF7 | 49.097 | 277 | 121 | 5 |
| G5190.t1 | Q8Y0J2 | 31.086 | 267 | 155 | 8 |
| G5190.t1 | G4NES9 | 28.676 | 272 | 172 | 8 |
| G5190.t1 | B9J010 | 29.181 | 281 | 159 | 10 |

Supplementary Table 7. Statistics of annotation results of CARD database.

| **ORF_ID** | **Best_Hit_ARO** | **ARO** | **AMR gene family** |
| --- | --- | --- | --- |
| g5185.t1 | catB7 | 3002679 | chloramphenicol acetyltransferase (CAT) |
| g5186.t1 | ermB | 3000375 | Erm 23S ribosomal RNA methyltransferase |
| g5187.t1 | abcA | 3003942 | ATP-binding cassette (ABC) antibiotic efflux pump |
| g5188.t1 | vanD | 3000005 | glycopeptide resistance gene cluster; van ligase |
| g5189.t1 | baeS | 3000829 | resistance-nodulation-cell division (RND) antibiotic efflux pump |
| g5190.t1 | arlS | 3000839 | major facilitator superfamily (MFS) antibiotic efflux pump |
| g5191.t1 | adeB | 3000775 | resistance-nodulation-cell division (RND) antibiotic efflux pump |
| g5192.t1 | FPH-1 | 3004795 | FPH beta-lactamase |
| g5193.t1 | tet(G) | 3000174 | major facilitator superfamily (MFS) antibiotic efflux pump |
| g5194.t1 | taeA | 3003986 | ATP-binding cassette (ABC) antibiotic efflux pump |

Supplementary Table 8. Statistics of annotation results of CYP450.

| **Database** | **Number** |
| --- | --- |
| CYP450 | 1,366 |

Supplementary Table 9. Statistics of annotation results of DFVF database.

| **Gene_ID** | **VFDB_ID** | **Description** |
| --- | --- | --- |
| g11007.t1 | SPCA_ARTOC | Carboxypeptidase S1 homolog A |
| g11324.t1 | SED1_ASPFU | Tripeptidyl-peptidase sed1 |
| g12139.t1 | DPP5_ARTBC | Probable dipeptidyl-peptidase 5 |
| g12201.t1 | SPCA_ARTOC | Carboxypeptidase S1 homolog A |
| g136.t1 | CXT1_CRYNJ | Beta-1,2-xylosyltransferase 1 |
| g1697.t1 | CTSD_ASPFU | Aspartic-type endopeptidase ctsD |

Supplementary Table 10. Statistics of annotation results of TCDB database, SignalP and tmhmm software.

| **Software/Database** | **Protein_type** | **Number** |
| --- | --- | --- |
| TCDB | Membrane transport protein | 2,139 |
| signalP | Signal peptide protein | 1,182 |
| tmhmm | Transmembrane protein | 2,729 |
| tmhmm | Secreted protein | 926 |

Supplementary Table 11. Statistics on Software and Database.

| **Software/Database** | **Version** | **Website** |
| --- | --- | --- |
| genomescope | 1.0.0 | <https://github.com/schatzlab/genomescope> |
| NECAT | 0.0.1_20200119 | <https://github.com/xiaochuanle/NECAT> |
| nextDenovo | 2.3.1 | <https://github.com/Nextomics/NextDenovo> |
| Racon | 1.4.13 | <https://github.com/isovic/racon> |
| Purge Haplotigs | 1.1.2 | <https://bitbucket.org/mroachawri/purge_haplotigs> |
| minimap2 | 2.17-r941 | <https://github.com/lh3/minimap2> |
| bwa | 0.7.17 | <https://github.com/lh3/bwa> |
| samtools | 1.10 | <https://github.com/samtools/samtools> |
| pilon | 1.23 | <https://github.com/broadinstitute/pilon> |
| purge_haplotigs | Latest Version | <https://github.com/skingan/purge_haplotigs_multiBAM> |
| braker | 2.1.5 | <https://github.com/Gaius-Augustus/BRAKER> |
| BUSCO | 4.1.4 | <https://github.com/RoyNexus/busco> |
| INFERNAL | 1.1.3 | <https://github.com/EddyRivasLab/infernal> |
| RepeatModeler | 1.0.4 | <https://github.com/Dfam-consortium/RepeatModeler> |
| RepeatMasker | 4.0.7 | <https://github.com/rmhubley/RepeatMasker> |
| BLAST+ | 2.9.0 | <https://github.com/enormandeau/ncbi_blast_tutorial> |
| Diamond | 2.0.4 | <https://github.com/bbuchÑnk/diamond> |
| KOBAS | 3.0 | <https://github.com/xmao/kobas> |
| HMMER | 3.2.1 | <https://github.com/EddyRivasLab/hmmer> |
| SignalP | 5.0 | <http://www.cbs.dtu.dk/services/SignalP/> |
| tmhmm | 2.0 | <http://www.cbs.dtu.dk/services/TMHMM/> |
| InterProScan | 5.33-72.0 | <https://github.com/ebi-pf-team/interproscan> |
| Uniport | 2019_07 | <https://www.uniprot.org/> |
| refseq | Latest version | <https://www.ncbi.nlm.nih.gov/refseq/> |
| Pfam | 31.0 | <https://pfam.xfam.org/> |
| GO | 2014 | <http://geneontology.org/> |
| KEGG | 87.0-20180701 | <https://www.kegg.jp/kegg/> |
| COG | 2014 | <https://www.ncbi.nlm.nih.gov/COG/> |
| Nr | 201909 | <https://ftp.ncbi.nlm.nih.gov> |
| Rfam | Latest version | <http://rfam.xfam.org/> |
| CAZy | Latest version | <http://www.cazy.org/> |
| PHI | Latest version | <http://www.phi-base.org> |
| CARD | Latest version | <https://card.mcmaster.ca/> |
| CYP450 | Latest version | <http://drnelson.utmem.edu/CytochromeP450.html> |
| DFVF | Latest version | <http://sysbio.unl.edu/DFVF/> |
| TCDB | Latest version | <http://www.tcdb.org/> |
| antiSMASH | 6.1.1 | <https://docs.antismash.secondarymetabolites.org/> |
| GNPS | 1.3.16 | <https://gnps.ucsd.edu/> |

Supplementary Table 12. antiSMASH and MN analyze information about SMs.

| **No.** | **Compound name** | **Source** | **Biological activity** | **GenBank** | **References** |
| --- | --- | --- | --- | --- | --- |
| A-1 | neosartorin | *Aspergillus novofumigatus* IBT 16806 | Antibacterial | MSZS01000005.1 | 2 |
| A-2 | nidulanin A | *Aspergillus nidulans* FGSC A4 | / | BN001308.1 | 3 |
| A-3 | asperlactone | *Aspergillus ochraceus* | Antimicrobial | AY540947.1 | 4 |
| A-4 | squalestatin | *Aspergillus* sp. Z5 | Antifungal | LDZW01000177.1 | 5 |
| A-5 | penicillin | *Penicillium chrysogenum* | Antibacterial | DQ192518.1 | 6 |
| A-6 | fellutamide B | *Aspergillus nidulans* FGSC A4 | Proteasome inhibitor | BN001302.1 | 7 |
| A-7 | equisetin | *Fusarium heterosporum* | Antibacterial | KC439347.1 | 8 |
| A-8, G-4 | destruxin A | *Metarhizium robertsii* ARSEF 23 | Insecticidal; Anticancer; Anti-osteoporosis | GL698760.1 | 9 |
| G-1 | / | */* | / | / | / |
| G-2 | Khellin | *Aspergillus versicolor* | / | / | 10 |
| G-3 | / | */* | / | / | / |
| G-5 | gymnodimine | *Karenia selliformis* | Toxins | / | 11 |
| G-6 | carabrol | *Carpesium abrotanoides* | Anticancer, antiviral | / | 12 |
| G-7 | 4-hydroxyvertixanthone | *Aspergillus wentii* | α-Glucosidase inhibitory activity | / | 13 |
| G-8 | elvitegravir | */* | HIV-1 integrase inhibitors | / | 14 |

ITS gene sequence

TGGGATCCTACTGATCCGAGGTCACCTGAAGAAAAATGGTTGGAGACGTCGGCTGGCGCCCGGCCGGCCCTAGTCGAGCGGGTGACAAAGCCCCATACGCTCGAGGACCGGACACGGTGCCGCCGCTGCCTTTCGGGCCCGTCCCCCGGGGGGGACGACGACCCAACACACAAGCCGGGCTTGATGGGCAGCAATGACGCTCGGACAGGCATGCCCCCCGGAATGCCAGGGGGCGCAATGTGCGTTCAAAGACTCGATGATTCACTGAATTCTGCAATTCACATTACTTATCGCAGTTCGCTGCGTTCTTCATCGATGCCGGAACCAAGAGATCCATTGTTGAAAGTTTTGACTGATTTTATATTCAGACTCAGACTGCATCACTCTCAGGCATGAAGTTCAGTAGTCCCCGGCGGCTCGCCCCCGAGGGGGTTCCCCGCCGAAGCAACAGTGTTAGGTATTCACGGGTGGGAGGTTGGGCGCCCGGAGGCAGCCCGCACTCAGTAATGATCCTTCCGCAGGTTCCCCTTTACGGA

Culture medium

Rice-solid Medium: rice 80 g , water 120 mL, per 1 L flask.

Czapek-Dox Medium: NaNO_3_ 3.0 g L^-1^, K_2_HPO_4_ 1.0 g L^-1^, MgSO_4_·7H_2_O 0.5 g L^-1^, KCl 0.5 g L^-1^, FeSO_4_ 0.01 g L^-1^, Sucrose 30.0 g L^-1^.

Potato Dextrose Broth (PDB): Potato 200 g L^-1^, Glucose 20 g L^-1^.

Potato Dextrose Agar (PDA): Potato 200 g L^-1^, Glucose 20 g L^-1^, Agar 15-20 g L^-1^.

Luria-Bertani (LB) medium: Tryptone g L^-1^, Yeast extract 5 g L^-1^, NaCl 10 g L^-1^, Agar 15-20 g L^-1^.

References

1. Kim D, Gilchrist CLM, Chun J, Steinegger M. UFCG database of universal fungal core genes and pipeline for genome-wide phylogenetic analysis of fungi. Nucleic Acids Res. 2023; 51 (D1): D777-D784.

http://doi.org/[10.1093/nar/gkac894](https://doi.org/10.1093/nar/gkac894)

1. Matsuda Y, Gotfredsen CH, Larsen TO. Genetic characterization of neosartorin biosynthesis provides insight into heterodimeric natural product generation. Org Lett. 2018; 20 (22): 7197-7200.

<https://doi.org/10.1021/acs.orglett.8b03123>

1. Andersen MR, Nielsen JB, Klitgaard A, Petersen LM, Zachariasen M, Hansen TJ, et al. Accurate prediction of secondary metabolite gene clusters in filamentous fungi. Proc Natl Acad Sci U.S.A. 2012; 110 (1): E99-E107.

<https://doi.org/10.1073/pnas.1205532110>

1. Bacha N, Dao HP, Atoui A, Mathieu F, O'Callaghan J, Puel O, et al. Cloning and characterization of novel methylsalic-ylic acid synthase gene involved in the biosynthesis of isoasperlactone and asperlactone in *Aspergillus westerdijkiae.* Fungal Genet Biol. 2009; 46 (10): 742-749.

<https://doi.org/10.1016/j.fgb.2009.07.002>

1. Bonsch B, Belt V, Bartel C, Duensing N, Koziol M, Lazarus CM, et al. Identification of genes encoding squalestatin S1 biosynthesis and in vitro production of new squalestatin analogues. Chem Commun. 2016; 52 (41): 6777-6780.

<https://doi.org/10.1039/C6CC02130A>

1. Fierro F, García-Estrada C, Castillo NI, Rodríguez R, Velasco-Conde T, Martín JF, et al. Transcriptional and bioinformatic analysis of the 56.8kb DNA region amplified in tandem repeats containing the penicillin gene cluster in *Penicillium chrysogenum.* Fungal Genet Biol. 2006; 43 (9): 618-629.

<https://doi.org/10.1016/j.fgb.2006.03.001>

1. Yeh HH, Ahuja M, Chiang YM, Oakley CE, Moore S, Yoon O, et al. Resistance gene-guided genome mining: serial promoter exchanges in *Aspergillus nidulans* reveal the biosynthetic pathway for fellutamide B, a proteasome inhibitor. ACS Chem Biol. 2016; 11 (8): 2275-2284.

<https://doi.org/10.1021/acschembio.6b00213>

1. Kakule TB, Sardar D, Lin Z, Schmidt EW. Two related pyrrolidinedione synthetase loci in *fusarium heterosporum* ATCC 74349 produce divergent metabolites. ACS Chem Biol. 2013; 8 (7): 1549-1557.

<https://doi.org/10.1021/cb400159f>

1. Wang B, Kang Q, Lu Y, Bai L, Wang C. Unveiling the biosynthetic puzzle of destruxins in *Metarhizium* species. Proc Natl Acad Sci U.S.A. 2012; 109 (4): 1287–1292.

<https://doi.org/10.1073/pnas.1115983109>

1. Shen SY, Xiong W, Li SS, Liu XS, Li YK, Miao D, et al. Chromones from the *tobacco* derived fungus *Aspergillus versicolor* and their antiviral activity. Chem Nat Compd+. 2023; 59 (3): 462-466.
2. Truman P, Stirling DJ, Northcote P, Lake RJ, Hannah DJ. Determination of brevetoxins in shellfish by the neuroblastoma assay. J AOAC Int. 2002; 85 (2002): 1057-1063.

<https://doi.org/10.1093/jaoac/85.5.1057>

1. Wang JF, He WJ, Zhang XX, Zhao BQ, Liu YH, Zhou XJ, et al. Dicarabrol, a new dimeric sesquiterpene from *Carpesium abrotanoides L.* Bioorg Med Chem Lett. 2015; 25 (19): 4082-4084.

<https://doi.org/10.1016/j.bmcl.2015.08.034>

1. Ma TT, Shan WG, Ying YM, Ma LF, Liu WH, Zhan ZJ. Xanthones with α-glucosidase inhibitory activities from *Aspergillus versicolor*, a fungal endophyte of *Huperzia serrata.* Helv Chim Acta. 2015; 98 (1): 148-152.

<https://doi.org/10.1002/hlca.201400165>

1. Sato M, Motomura T, Aramaki H, Matsuda T, Yamashita M, Ito Y, et al. Novel HIV-1 integrase inhibitors derived from quinolone antibiotics. J Med Chem. 2006; 49 (5): 1506-1508.

<https://doi.org/10.1021/jm0600139>
